# Shared drone route scheduling optimization

**DOI:** 10.1371/journal.pone.0348883

**Published:** 2026-05-19

**Authors:** Chao Hong, Yi Yan, Zhigang Lian, Xiangrong Li

**Affiliations:** 1 School of Electronic and Information Engineering, Shanghai DianJi University, Shanghai, China; 2 China-ASEAN School of Economics, Guangxi University, Nanning, Guangxi, China; Southwest Jiaotong University, CHINA

## Abstract

Shared passenger-carrying unmanned aerial vehicle (UAV) systems offer a promising solution for urban air mobility, yet their real-time scheduling in multi-airport environments remains challenging due to heterogeneous passenger demands, model compatibility constraints, and the need to balance efficiency with service quality. This study addresses these challenges by proposing a shared UAV route scheduling optimization model for multi-airport, multi-station systems. The model integrates passenger order characteristics (origin, destination, party size, preferred UAV type, and desired arrival time) with UAV operational parameters (current location, remaining endurance, seating capacity, speed, and model), and incorporates a quadratic soft time window mechanism to flexibly manage arrival deviations while minimizing total system navigation time. To solve this discrete combinatorial optimization problem, we develop a self-learning Ant-Lion Optimizer (SLALO) with natural number encoding that dynamically tracks seat availability and updates route assignments in real-time. Simulation results demonstrate that the proposed approach achieves a 27.3% reduction in total navigation time compared to non-pooling baselines, with an average UAV utilization rate of 78.6%. Comparative analysis against Genetic Algorithm (GA), Particle Swarm Optimization (PSO), and standard Ant-Lion Optimizer (ALO) shows that SLALO achieves superior convergence speed and solution quality. The proposed framework is particularly relevant for border regions with limited ground infrastructure, where intelligent shared mobility systems can serve as a critical driver for cultivating new quality productive forces and supporting regional economic integration. These findings highlight the potential of shared UAV systems to improve urban air mobility efficiency, reduce operational costs, and enhance passenger satisfaction.

## Introduction

Urban transportation systems worldwide are facing increasing pressure from congestion, limited infrastructure, and environmental concerns. Urban Air Mobility (UAM) has emerged as a promising solution to alleviate ground traffic by leveraging passenger-carrying UAVs [1]. Unlike traditional ground transport, UAVs offer flexibility, reduced travel time, and the potential for on-demand services. However, most existing research focuses on cargo drone routing, often modeled as vehicle routing problems (VRP), which do not fully capture the complexities of passenger transport[2]. Passenger-carrying UAV scheduling must consider time constraints, ride-pooling coordination, model compatibility, and multi-airport coordination [3].

While these studies have established foundational methodologies for path planning and fleet coordination, they typically assume homogeneous vehicle fleets, static demand, and rigid time windows. In the context of passenger transport, several critical gaps remain. First, passenger-carrying UAV operations require explicit consideration of model compatibility, as users may have preferences or constraints regarding aircraft type. Second, ride-pooling coordination—a key mechanism for improving system efficiency—demands dynamic seat availability tracking and real-time route adjustments, which are not adequately addressed in conventional VRP formulations. Third, multi-airport systems introduce additional complexity in fleet allocation and return-to-base constraints that are often simplified in single-depot models. Furthermore, the integration of passenger time preferences with operational efficiency remains underexplored, with most studies adopting either rigid time windows that sacrifice flexibility or ignoring time constraints altogether.

This study is particularly motivated by the unique transportation challenges in China’s border regions, including Guangxi, Yunnan, Xinjiang, Tibet, Inner Mongolia, Heilongjiang, Jilin, and Liaoning. These areas are characterized by vast geographical expanse, low population density, complex terrain, and relatively underdeveloped ground transportation infrastructure. Conventional road-based transport often suffers from long travel times, high costs, and limited accessibility in remote border communities. Urban Air Mobility (UAM), enabled by shared passenger-carrying UAVs, offers a transformative solution to bridge these spatial gaps, improve connectivity, and support economic integration in border areas. Therefore, while the proposed model is generally applicable, its practical significance is especially pronounced in the context of China’s border regions, where the development of new quality productive forces in transportation can serve as a critical driver for regional economic revitalization.

Despite advancements in multi-depot routing and swarm coordination, limited attention has been given to real-time shared UAV scheduling in multi-airport systems with heterogeneous passenger demands[4]. Rigid time windows often reduce operational flexibility, while ignoring time preferences may compromise service quality. To address this gap, this study proposes a balanced scheduling model that incorporates both efficiency and passenger time satisfaction. A soft time window mechanism with quadratic penalties is introduced to manage arrival deviations flexibly.

The main contributions of this study are threefold:

(1) A multi-airport shared passenger UAV scheduling model is formulated, incorporating endurance, capacity, and model compatibility constraints.
(2) A quadratic soft time window mechanism is integrated to balance operational efficiency and passenger satisfaction.
(3) A discrete encoding strategy within the SLALO framework is developed to solve large-scale combinatorial assignment problems efficiently.

Passenger Demand-Based Total Time Minimum Route Planning Model for Multi-Airport Systems

### 3.2. System description

This paper studies the shared UAV commuting system aiming to determine a passenger-carrying UAV flight scheduling scheme based on multiple airports satisfying different passenger needs, capable of meeting various real-life scenarios. In the total time-minimum route planning model for multi-airport systems based on different passenger needs, we envision an ideal scenario with multiple airports, multiple drone sites, and each user with corresponding needs. When users initiate flight orders to the cloud server, passenger-carrying UAVs upload their site locations. The model must deploy a UAV meeting user needs while maximizing utilization. The primary computational objective is to minimize the total voyage time of all UAVs in the system.

It is assumed that multiple UAV airports in the region can assign UAVs meeting user model, passenger count, and time requirements to the user’s home station, and UAVs have unloaded mileage loss. When a passenger initiates a boarding order at a certain location and time, specifying arrival time, the system responds, allocates a UAV to depart from the airport to the starting station, transports the passenger to the target station along the route, and returns to the airport. Our shared UAV commuting system (SUCS) rationally allocates UAV tasks to minimize total system time.

According to model assumptions, passenger-carrying UAVs uniformly depart from respective airports for maiden flights and continuously match demand points and carpool-able drones while orders are executed; all order and drone information is shared during flights; the system operates in real-time scheduling, with users specifying arrival times for their demands.

**Fig 1:**
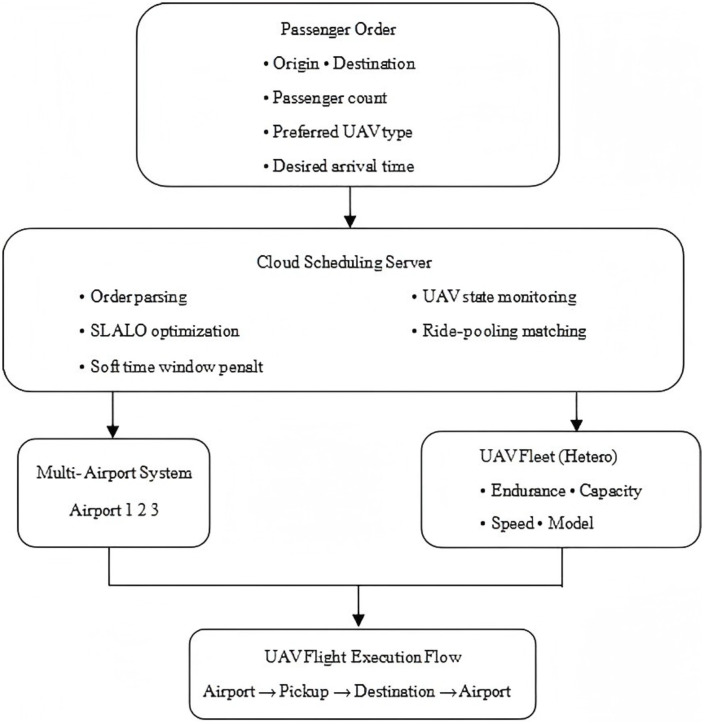
Schematic diagram of the shared UAV scheduling framework.

To illustrate the system operation process, Fig 1 presents a schematic diagram of the shared UAV scheduling framework organized in four hierarchical layers. The first layer depicts passenger orders, each specifying origin, destination, passenger count, preferred UAV model, and desired arrival time. These orders are transmitted to the second layer, the cloud scheduling server, which integrates multiple functional modules: order parsing, UAV state monitoring, SLALO-based optimization, ride-pooling matching, and soft time window penalty calculation. The server then coordinates with the third layer, which consists of two interconnected components: the multi-airport system (Airports 1, 2, 3) where UAVs are stationed, and the heterogeneous UAV fleet characterized by varying endurance, capacity, speed, and model specifications. The final layer illustrates the UAV flight execution flow: each assigned UAV departs from its airport, proceeds to the passenger’s origin site for pick-up, transports the passenger(s) to the destination site, and returns to an airport upon mission completion. This hierarchical structure reflects the end-to-end scheduling process and the key decision-making modules embedded in the proposed model.

### 3.2. Model assumptions

To solve the above scheduling problem, a mathematical model is developed with the following assumptions:

(1) Passenger demand is fixed once the order is submitted. The number of passengers associated with each request does not change during scheduling;
(2) Each passenger order is served by only one UAV and cannot be transferred. This assumption ensures service continuity and simplifies scheduling, as passengers are not required to change aircraft during their journey;
(3) UAV cruising speed is model-dependent and remains constant during flight segments. Speed variations caused by weather or air traffic control are not considered;
(4) Take-off and landing times are not included in the cruising speed but are incorporated as fixed time penalties added to the total navigation time for each mission segment;
(5) Influences such as inclement weather, airframe failure, and acoustic and visual interference are not considered;
(6) All UAVs depart from the airport and return to the airport upon mission completion.

The model concepts are defined as follows.

Passenger demand definition: Passenger demand $pr_{c}=\left(O,E,p,S_{c},t\right)$, where O is the initiation Site, *E* is the destination site, *p* is the number of passeng*ers*，*S*_*C*_ is the aircraft type requirement of passenger *C*, and *t* is the time when the passenger needs to arrive.

Shared passenger-carrying UAV. Set Definition: Shared collection of passenger-carrying drones $UAV=\left(d_{\text{1}},d_{\text{2}},...,d_{U}\right)$, For any *drone*_*u*_ ∈ *UAV,* The following status information is available, $d_{u}=\left(L,r,S_{u},z,v\right)$, where *L* is the current location of the drone, *r* is the range of the drone, *S*_*u*_ is a model of the UAV U, *z* is the number of seats, *v* is drone speed.

Definition of drone airports: aerodrome $AIRPORT=\left(a_{\text{1}},a_{\text{2}},...,a_{M}\right)$, where *a*_*m*_∈*AIRPORT* is the location coordinates of the drone field.

Drone site definition: Site $STATION=\left(s_{\text{1}},s_{\text{2}},...,s_{N}\right)$, where *s*_*i*_∈*AIRPORT* is the location coordinates of the drone site.

Airport vs. Site Distinction: Airports are facilities where UAVs are stationed, maintained, and to which they must return after missions. Sites are passenger pick-up and drop-off locations distributed throughout the service area. UAVs always start and end at airports, while serving passenger requests at sites in between.

### 3.3. Mathematical models

In order to describe the model more clearly and accurately, the definition symbols are as follows:

M: Total number of drone airports, m: UAV airport serial number；

N: total number of UAV sites, i,j: They represent the serial numbers of the drone sites；

U: Total number of drones； u: Drone serial number；

C: Total number of passengers； c: Passenger serial number；

$\overset{u}{\underset{mi}{x}}$: The Boolean logical value  u of the drone from the airport to the site  m, which is 1 if the drone  i passes through the airport   u to the site m, otherwise it is 0  i;

$\overset{𝐮}{\underset{𝐢𝐣}{𝐱}}$: Boolean logical value  u of the drone  i from site j  to site;

$\overset{u}{\underset{jm}{x}}$: Boolean logical value  u of the drone  j from site m to site;

$d_{mi}$: Distance from airport  m to site i;

$d_{ij}$: i Site-to-site distance j;

$d_{jm}$: Distance from airport  j to site  m；

$v_{u}$: The speed of  u the drone；

$r_{u}$: The cruising range of the  u drone；

$B_{u}$: Battery safety margins for UAV u；

$\overset{c}{\underset{ij}{S}}$: Passenger c specifies the drone model requirements from site i to site j；

$\overset{u}{\underset{ij}{S}}$: The order assigns the model of drone u from site i to site  j;

$\overset{c}{\underset{ij}{P}}$: The number of passengers c required to travel from station i to station j;

$\overset{u}{\underset{ij}{Z}}$: remaining seats of drone u when departing from site i to site j (initialized as the drone’s total seating capacity; after picking up passengers, the remaining seats are reduced accordingly; upon drop-off, seats become available again for subsequent segments);

$\overset{c}{\underset{ij}{T}}$: Passenger c specifies a time requirement for arriving at station j The passenger departs from station i;

$\overset{u}{\underset{ij}{T}}$: The time for an order-assigned UAV u to arrive at site j after departing from site i;

Based on the above definitions and assumptions, the SUCS model based on minimizing the total time of the multi-airport UAV operation system is designed as shown below:


$$\begin{array}{c} min\left\{\sum_{m=1}^{m=M}\sum_{i=1}^{i=N}\sum_{u=1}^{U}\overset{u}{\underset{mi}{x}}\;d_{mi}/v_{u}+\sum_{i=1}^{i=N}\sum_{j=1}^{j=N}\sum_{u=1}^{U}\overset{u}{\underset{ij}{x}}\;d_{ij}/v_{u}+\sum_{m=1}^{m=M}\sum_{j=1}^{j=N}\sum_{u=1}^{U}\overset{u}{\underset{jm}{x}}\;d_{jm}/v_{u}\right\} \end{array}$$ (1)

$$\begin{array}{c} r_{u}>\sum_{m=1}^{m=M}\sum_{i=1}^{i=N}\overset{u}{\underset{mi}{x}}d_{mi}+\sum_{i=1}^{i=N}\sum_{j=1}^{j=N}\overset{u}{\underset{ij}{x}}d_{ij}+\sum_{m=1}^{m=M}\sum_{j=1}^{j=N}\overset{u}{\underset{jm}{x}}d_{jm}+B_{u}\; \end{array}$$ (2)

$$\begin{array}{c} \overset{c}{\underset{ij}{S}}\leq \overset{\mu }{\underset{ij}{S}} \end{array}$$ (3)

$$\begin{array}{c} \overset{c}{\underset{ij}{P}}\leq \overset{u}{\underset{ij}{Z}} \end{array}$$ (4)

$$\begin{array}{c} \overset{c}{\underset{ij}{T}}\geq \overset{u}{\underset{ij}{T}} \end{array}$$ (5)

Eq. (1) means that the ultimate goal of the model is to minimize the total navigation time of all UAVs; constraint [2] means that the remaining endurance of the assigned UAV corresponding to the task must meet the needs of the task; constraint [3] means that the assigned UAV model must meet or exceed the passenger’s requested model (allowing upgrades to improve fleet utilization); constraint [4] means that the system assigns the UAVs to have unloaded seats inside the UAVs that can accommodate all of the passengers of the order; and constraint [5] means that the UAVs need to send the passengers to their destinations at an earlier time than the passengers’ demanded arrival times.

## 3. A soft time window based minimum route planning model for total consumption of multi-airport systems

### 3.1 Description of the problem

In real life, some passengers have restrictions on arrival time (e.g., to arrive at airport, train station within a certain time). If a new order with high carpool adaptability emerges, forcing carpooling might reduce total flight distance but could seriously impact personal interests of order initiators. Therefore, from the passenger’s time requirements perspective, violations should incur penalties.

The Soft Time Window[5] based Total Consumption Minimum Route Planning Model [6] for Multi-Airport Systems builds on the scheduling problem and guarantees passenger interests by adding time constraints for UAV arrival at passenger locations [7]. If SUCS scheduling time deviates from passenger order initiation time, penalty cost is added accordingly.

### 3.2 Modeling of penalties for exceeding demand time

We introduce asymmetric quadratic penalty functions to appropriately reflect passenger disutility:


$$\begin{array}{c} \begin{array}{c} \overset{u}{\underset{ij}{f}}\left\{ \begin{array}{c} @l@ec(\overset{c}{\underset{ij}{T}}-\overset{u}{\underset{ij}{T}})\text{\textbackslash{}hspace\{0.33em}\;\;\;\}\;\text{\textbackslash{}hspace\{0.33em}\;\}\overset{c}{\underset{ij}{T}}>\overset{u}{\underset{ij}{T}}\\ 0\text{\textbackslash{}hspace\{0.33em}\;\;\;\;\;\;\;\;\;\;\;\;\;\;\;\}\;\text{\textbackslash{}hspace\{0.33em}\;\}\;\;\;\overset{c}{\underset{ij}{T}}=\overset{u}{\underset{ij}{T}}\\ \text{\textbackslash{}hspace\{0.33em}\}\\ lc(\overset{u}{\underset{ij}{T}}-\overset{c}{\underset{ij}{T}})\text{\textbackslash{}hspace\{0.33em}\;\;\;\;\;\}\;\text{\textbackslash{}hspace\{0.33em}\}\overset{c}{\underset{ij}{T}}<\overset{u}{\underset{ij}{T}} \end{array}\right.\;\\ \;\;\;\;\;\;\;\;\;\;\;\;\;\;\;\;\;\; \end{array} \end{array}$$ (6)

included among these,

$\overset{u}{\underset{ij}{f}}$: The value of time loss (TIME COST) of UAV u from site i to site j;

*ec*: Systems Penalty for Early Order Fulfillment for passenger-carrying Drones；

*lc*: Systematic penalties for passenger-carrying drones that fail to fulfill orders on time and in accordance with the time specified by the user；

Justification for quadratic form: Quadratic penalties better reflect passenger dissatisfaction with extreme delays; small deviations cause minor inconvenience, while large delays cause disproportionate dissatisfaction. Early penalties are justified because passengers may not be ready for early pick-up, early arrivals occupy limited waiting areas, consume battery while hovering, and may disrupt airspace slots.

### 3.3. mathematical model

To describe the model more clearly and accurately, the notation is defined as follows:

*α*_*μ*_: distance cost coefficient for UAV u (RMB/km)

*β*: normalization factor converting time penalty to cost units (RMB/min)

The other notations of the model are the same as in 2.2.

The distance cost coefficient *α*_*μ*_ is specified in RMB per kilometer (as listed in Tables 1, 2, and 3 for each UAV model). The normalization factor *β* converts time penalties (minutes) into cost units (RMB), ensuring dimensional consistency between distance-related and time-related terms in the objective function. In this study, *β* is set to 1 RMB/min based on typical urban mobility valuation benchmarks, reflecting the trade-off between operational distance costs and passenger time satisfaction. Both parameters thus share consistent units, allowing the objective function to combine distance and time components meaningfully.

**Table 1:** UAV airport information.

The drone airport number	location
1	(55,55)
2	(41,61)
3	(74,74)

**Table 2:** UAV information.

Drone number	Endurance/m	Average speed/m/min	Number of seats	Model	Required battery margin/m	Billing/RMB
1	300000	1500	2	standard	2000	10 + 6/km
2	300000	1500	2	standard	2000	10 + 6/km
3	300000	1500	2	standard	2000	10 + 6/km
4	300000	1500	2	standard	2000	10 + 6/km
5	300000	1500	2	standard	2000	10 + 6/km
6	300000	1500	2	standard	2000	10 + 6/km
7	300000	1500	2	standard	2000	10 + 6/km
8	300000	1500	2	standard	2000	10 + 6/km
9	300000	1500	2	standard	2000	10 + 6/km
10	300000	1500	2	standard	2000	10 + 6/km

**Table 3:** UAV information.

Drone number	Endurance/m	Average speed/m/min	Number of seats	Model	Required battery margin/m	Billing/RMB
11	300000	1833	2	express	2000	14 + 8/km
12	300000	1833	2	express	2000	14 + 8/km
13	300000	1833	2	express	2000	14 + 8/km
14	300000	1833	2	express	2000	14 + 8/km
15	300000	1833	2	express	2000	14 + 8/km
16	300000	1833	2	express	2000	14 + 8/km
17	300000	1833	2	express	2000	14 + 8/km
18	300000	1833	2	express	2000	14 + 8/km
19	300000	2167	1	Plane	2000	18 + 16/km
20	300000	2167	1	Plane	2000	18 + 16/km

Based on the above assumptions and parameter definitions, the model is developed as follows:


$$\begin{array}{c} min\left\{\sum_{i=1}^{i=N}\sum_{j=1}^{j=N}\sum_{u=1}^{U}\overset{u}{\underset{ij}{x}}\;d_{ij}*\alpha_{u}\text{+}\sum_{i=1}^{i=N}\sum_{j=1}^{j=N}\sum_{u=1}^{U}\overset{u}{\underset{ij}{x}}\overset{u}{\underset{ij}{f}}*\beta \right\} \end{array}$$ (7)

$$\begin{array}{c} r_{u}>\sum_{m=1}^{m=M}\sum_{i=1}^{i=N}\overset{u}{\underset{mi}{x}}d_{mi}+\sum_{i=1}^{i=N}\sum_{j=1}^{j=N}\overset{u}{\underset{ij}{x}}d_{ij}+B_{u}\; \end{array}$$ (8)

$$\begin{array}{c} \overset{c}{\underset{ij}{S}}=\overset{u}{\underset{ij}{S}} \end{array}$$ (9)

$$\begin{array}{c} \overset{c}{\underset{ij}{P}}\leq \overset{u}{\underset{ij}{Z}} \end{array}$$ (10)

## Improved natural number encoding rules for SLALO algorithm

The SLALO algorithm is originally used to solve real number function problems, and in this paper, we obtain the encoding of the natural numbers for computing the discrete UAV route planning problem by rounding the real numbers. This subsection is mainly used to show how the system allocates the coding and decoding process for UAV service passengers, for example The specific transformation steps of SLALO for a passenger-carrying UAV are as follows:

It may be assumed that $A=\left[a_{\text{1}},a_{\text{2}},\cdots a_{j},\cdots ,a_{N}\right]\left(j=1,2,\cdots n\right)$ is a random ant lion.


$$\begin{array}{c} S=ceil\left(A\right) \end{array}$$ (11)

Ceil means to round A and find the absolute value to get S.

In the ant lion transformed by Eq. [11] $S=\left[s_{\text{1}},s_{\text{2}},\cdots s_{j},\cdots ,s_{N}\right]$, s1 represents the passenger with number 1, and the value of s1 indicates that the UAV with that number is going to serve that passenger.

The following shows the specific conversion flow for a string of SLALO codes with a user count of 8:

Using the coding transformation process of the appeal, the random real-number coded ant-lion individual A is changed from Eq. (11) to the SLALO feasible solution ant-lion individual S composed of the corresponding constants [8]. This ant-lion individual represents the following: Drone No. 6 serves both the users at the starting sites 1 and 3; the passengers at the starting sites 2, 4, and 6 are serviced by Drone No. 7; the passenger at location 5 is serviced by Drone No. 3; the passenger at location 7 is serviced by Drone No. 4; and the passenger at location 8 is serviced by Drone No. 1 (Tables 4 and 5).

**Table 4:** Real coding change to natural number coding for SLALO.

Ant lion/portion	1	2	3	4	5	6	7	8
*A*	5.03	6.49	5.968	6.61	2.11	6.43	3.54	0.75
*S*	6	7	6	7	3	7	4	1

Simulation experiment of UAV flight path optimization based on self-learning ant-lion algorithm

**Table 5:** Mission path of SUCS UAV in shared mode.

drone	Route	Mileage/m	Time/min
standard 1	s1→O12→O3→E3→E12→s1	24096.44	16.06
standard 2	s3→O2→E2→O10→O6→E10→E6→s3	24033.12	16.02
standard 3	s2→O7→O8→E7→E8→O9→O14→E9→E14→s2	22036.35	14.69
Express 4	s1→O1→O13→E13→E1→s1	22253.51	12.14
Express 5	s1→O11→O4→E11→E4→s1	21271.93	11.60
plane 6	s1→O5→E5→s1	24092.68	11.12

### 3.2. Drone flight path optimization case

The cloud server receives 14 orders with different starting and destination stations for different users. Table 6 contains the location of the start site and destination site for each passenger. The airport has several types of passenger-carrying drones, each with a uniform range of 300 km and models that can accommodate 1 and 2 passengers (see Tables 1 and 7 for UAV and airport details). The drones take off from the hangar center in 0 time and fly at the same speed as the average speed.

**Table 6:** The needs of passengers.

orders	Start site	Destination site	Specify the arrival time/min	Mileage/m
1	（77,67）	（25,54）	26	6200
2	（73,86）	（34,86）	30	4100
3	（46,23）	（42,46）	23	3300
4	（81,30）	（95,67）	27	5100
5	（86,17）	（47,95）	12	13000
6	（31,107）	（81,57）	34	9400
7	（46,42）	（11,17）	20	5800
8	（50,15）	（9,29）	24	6100
9	（11,50）	（31,67）	35	3900
10	（26,99）	（69,96）	56	5800
11	（69,31）	（62,92）	31	6900
12	（83,7）	（17,59）	13	13000
13	（75,35）	（75,35）	41	6300
14	（29,39）	（53,74）	70	5200

**Table 7:** UAV information.

Drone number	Endurance/m	Average speed/m/min	Number of seats	Model	Required battery margin/m	Billing/RMB
1	300000	1500	2	standard	2000	10 + 6/km
2	300000	1500	2	standard	2000	10 + 6/km
3	300000	1500	2	standard	2000	10 + 6/km
4	300000	1833	2	express	2000	14 + 8/km
5	300000	1833	2	express	2000	14 + 8/km
6	300000	2167	1	Plane	2000	18 + 16/km

**Fig 2:**
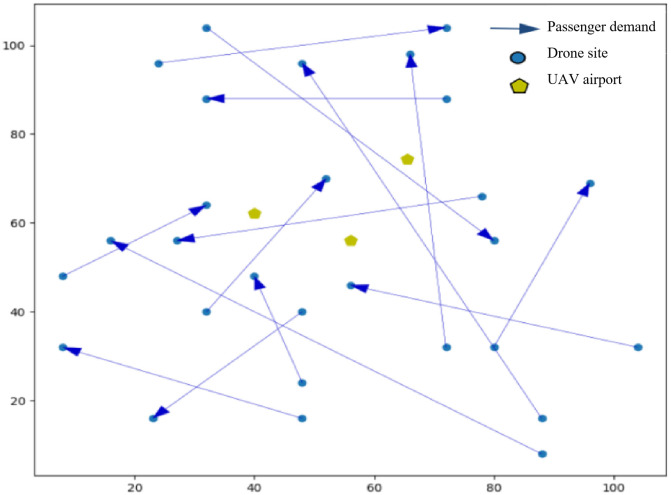
Passenger needs and airport location.

Fig 2 shows the spatial distribution of passenger origins and destinations.

### 3.4. Simulation experiment analysis

**Fig 3:**
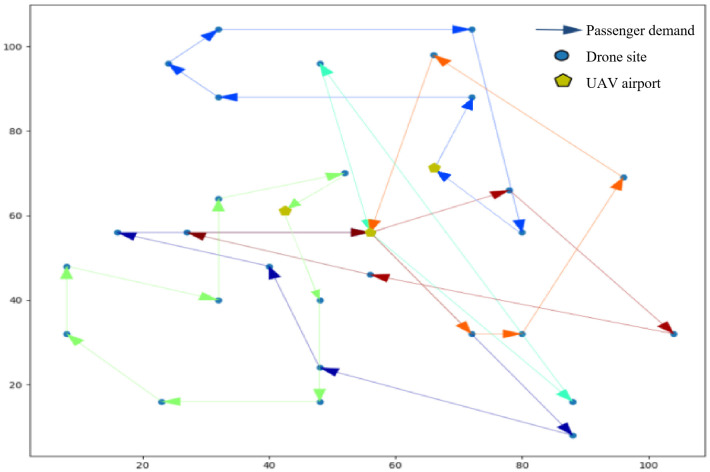
Minimum result of total time in multi-airport systems based on passenger needs.

**Fig 4:**
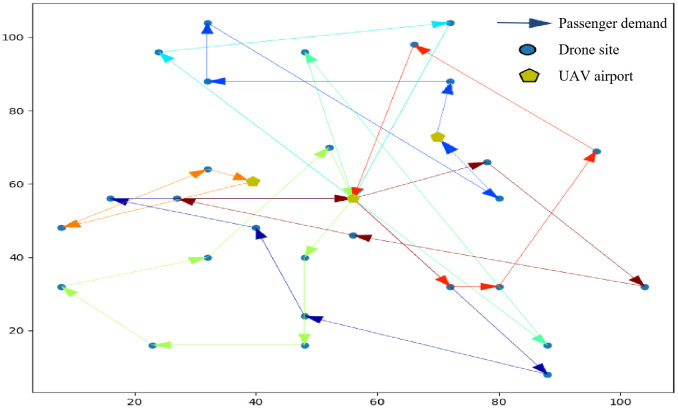
UAV route planning results under soft time window constraints (small-scale case).

Figs 3 and 4 show the UAV route planning graphs obtained from the simulation optimization experiments for the above mentioned small-scale cases, Fig 3 shows the total time minimum result of the multi-airport system based on passenger demand [9], and Fig 4 shows the total cost minimum result of the system based on the soft time window constraints.

The results show that 14 orders are served by 6 UAVs, with total voyage mileage 137,784 m and total operating time 81.63 min. In non-pooling mode, total distance would be 226.8 km, demonstrating a 27.3% reduction through ride-pooling. Average UAV utilization reaches 78.6%.

Comparison with other algorithms: To further validate the effectiveness of SLALO, we compared it with Genetic Algorithm (GA), Particle Swarm Optimization (PSO), and standard Ant-Lion Optimizer (ALO) on the large-scale case (50 orders, 20 UAVs, 3 airports). The detailed UAV information for drones 11–20 is provided in Table 3, and the three airport locations are listed in Table 8. Table 9 summarizes the performance metrics [10–15].

**Table 8:** UAV airport information.

The drone airport number	location
1	(500,500)
2	(200,200)
3	(800,800)

**Table 9:** The needs of passengers.

orders	Start site	Destination site	Specify the arrival time/min	Mileage/m
1	(400,810)	(260,671)	13	10000
2	(155,224)	(927,159)	23	6900
3	(318,789)	(211,198)	23	54300
4	(472,975)	(271,673)	8	32000
5	(791,401)	(220,361)	4	4000
6	(39,731)	(781,951)	6	23000
7	(79,966)	(619,610)	11	39000
8	(327,688)	(460,351)	7	33000
9	(359,951)	(899,739)	8	20300
10	(710,979)	(851,691)	12	31000
11	(829,891)	(661,91)	6	81000
12	(649,761)	(399,21)	19	78900
13	(610,299)	(551,791)	21	49400
14	(992,548)	(969,319)	14	22400
15	(721,251)	(969,120)	26	12000
16	(821,279)	(29,951)	19	66100
17	(658,869)	(9,221)	16	64100
18	(529,689)	(99,868)	6	17300
19	(429,180)	(29,632)	22	47900
20	(643,202)	(351,97)	17	9500
21	(842,71)	(39,241)	31	17100
22	(369,510)	(949,610)	21	9800
23	(318,629)	(421,933)	9	32100
24	(977,832)	(802,413)	5	36400
25	(523,717)	(378,681)	20	12100
26	(931,821)	(955,578)	22	21300
27	(691,567)	(981,63)	16	61000
28	(748,293)	(888,582)	23	30100
29	(619,62)	(382,621)	10	5230

**Fig 5:**
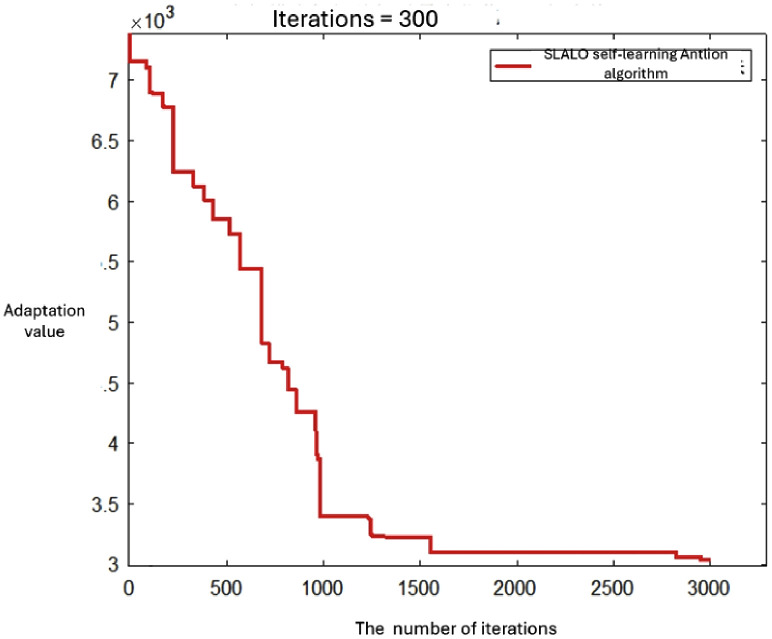
Convergence curves of GA, PSO, ALO, and SLALO for the large-scale case (total time minimization). The vertical axis labeled “Adaptation value” represents the objective function value, i.e., total navigation time (minutes).

**Fig 6:**
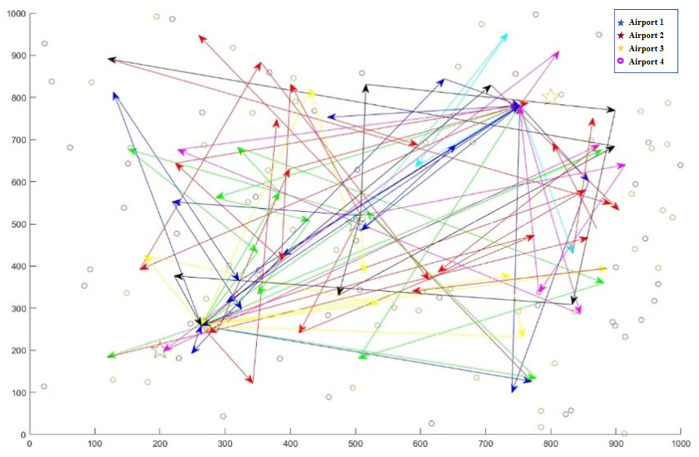
Large scale total time consuming minimum UAV allocation routes incorporating multi-airport issues.

**Fig 7:**
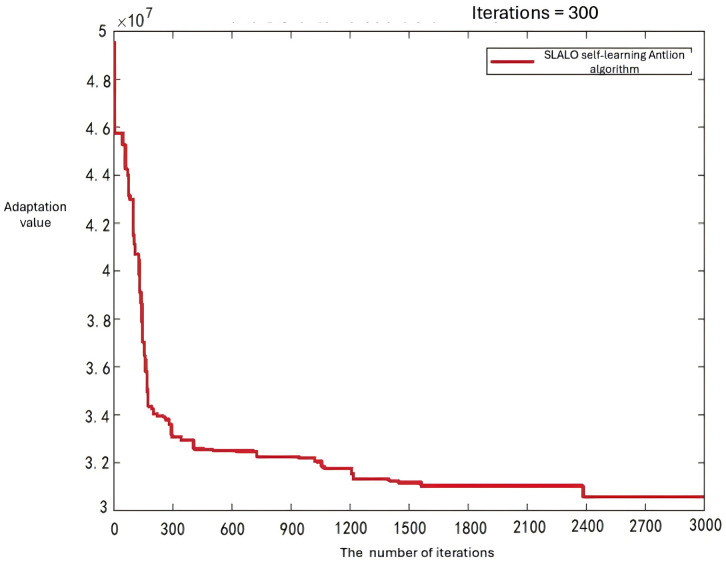
Convergence curves for total cost minimization under soft time window constraints (large-scale case). The vertical axis labeled “Adaptation value” represents the objective function value, i.e., total cost (RMB).

**Fig 8:**
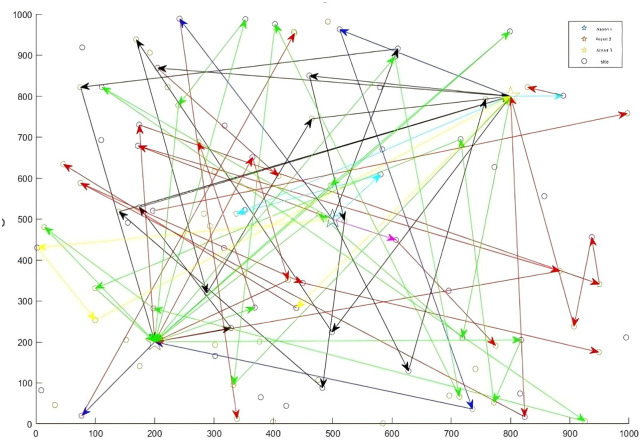
Large-scale total consumption drone allocation routes that include multiairport issues.

Fig 5 shows the convergence curves of the four algorithms for the large-scale case.  Fig 6–8 displays the optimal UAV routes for total cost minimization in the large-scale multi-airport system. Fig 7 shows the convergence curve for the total cost minimization (soft time window model) in the large-scale case (Fig 8).

Tables 2 and 10 detail the remaining passenger orders and standard UAV parameters (UAVs 1–10) for the large-scale case, while Tables 3 and 8 provide the specifications for additional UAVs [11–20] and the three airport locations, respectively.

**Table 10:** The needs of passengers.

orders	Start site	Destination site	Specify the arrival time/min	Mileage/m
30	(979,459)	(809,824)	2	32200
31	(663,900)	(152,792)	13	12300
32	(793,283)	(999,152)	18	9900
33	(287,759)	(948,95)	17	55000
34	(942,673)	(531,772)	25	12400
35	(293,853)	(973,400)	22	47700
36	(299,842)	(951,603)	1	23800
37	(828,372)	(863,503)	8	13100
38	(273,21)	(631,818)	7	81000
39	(810,22)	(399,389)	12	41200
40	(670,923)	(173,161)	10	72500
41	(57,1000)	(303,828)	7	17600
42	(283,579)	(2,7)	6	52900
43	(527,956)	(49,299)	24	64700
44	(574,712)	(27,525)	14	15400
45	(184,767)	(273,703)	7	5400
46	(950,412)	(314,672)	7	41200
47	(413,63)	(48,544)	8	51000
48	(889,298)	(39,270)	1	4100
49	(722,401)	(501,491)	6	7900
50	(807,227)	(887,364)	24	10900

## Conclusion

This paper addresses the shared passenger-carrying UAV scheduling problem in multi-airport systems by proposing a model that integrates passenger time preferences, UAV operational constraints (endurance, capacity, model compatibility), and ride-pooling coordination. A soft time window mechanism with quadratic penalties is introduced to balance operational efficiency and passenger satisfaction, allowing flexible yet controlled arrival deviations. To solve the resulting discrete combinatorial assignment problem, we develop a self-learning Ant-Lion Optimizer (SLALO) enhanced with natural number encoding.

The effectiveness of the proposed approach is demonstrated through simulation experiments. Key findings include: [1] the shared scheduling model achieves a 27.3% reduction in total navigation time compared to non-pooling baselines, with an average UAV utilization rate of 78.6%; [2] the SLALO algorithm outperforms GA, PSO, and standard ALO in terms of both convergence speed and solution quality; [3] the quadratic soft time window mechanism effectively balances cost efficiency and passenger time satisfaction. These results highlight the potential of shared UAV systems to improve urban air mobility efficiency, reduce operational costs, and enhance passenger satisfaction, particularly in regions with underdeveloped ground transportation infrastructure.

Beyond the immediate findings, the proposed shared UAV scheduling model holds significant promise for addressing transportation challenges in vast and remote border regions such as Tibet, Xinjiang, Inner Mongolia, and the northeastern provinces of China. In these areas, where ground infrastructure is limited and distances are substantial, the efficiency gains demonstrated in this study (27.3% reduction in navigation time) could translate into even more substantial socio-economic benefits. Future implementation of such systems could enhance cross-border connectivity, support emergency response in sparsely populated areas, and contribute to the balanced regional development objectives outlined in China’s border area revitalization strategies. From a broader perspective, the development of shared UAV-based mobility systems represents a concrete pathway for cultivating new quality productive forces in border regions, leveraging advanced automation and intelligent scheduling to overcome geographical constraints and unlock economic potential.

Future work will extend the model to dynamic demand scenarios and real-time re-scheduling under uncertainty, as well as incorporate more realistic factors such as weather effects and air traffic constraints.

## Supporting information

S1_Code This file contains the complete MATLAB simulation code of the SLALO (Shared Logistics Aerial Lift Optimization) algorithm adopted in this study. The code can reproduce the results presented in Table 5 of the manuscript. All required functions are compiled in a single PDF file, including the main script (main.m), objective function (UAV_objective.m), data loading (data_load.m), route generation (get_UAV_route.m), initialization (initialization_SLALO.m), mutation operator (Random_walk_around_antlion_SLALO.m), selection operator (RouletteWheelSelection_SLALO.m), visualization function (func_plot_SLALO.m), and the main SLALO optimization procedure (SLALO.m).
(PDF)
