# Supplementary material for: Shared drone route scheduling optimization
Source: PLoS One. 2026 May 19;21(5):e0348883. doi: 10.1371/journal.pone.0348883 (PMC13186358; doi:10.1371/journal.pone.0348883)
Supplement: S1_Code — The code can reproduce the results presented in Table 5 of the manuscript. All required functions are compiled in a single PDF file, including the main script (main.m), objective function (UAV_objective.m), data loading (data_load.m), route generation (get_UAV_route.m), initialization (initialization_SLALO.m), mutation operator (Random_walk_around_antlion_SLALO.m), selection operator (RouletteWheelSelection_SLALO.m), visualization function (func_plot_SLALO.m), and the main SLALO optimization procedure (SLALO.m). (PDF) [file pone.0348883.s001.pdf]

data_load.m

```
function [UAV_info, Passenger_info, Airport_info] = data_load(scene_type)

if nargin < 1
    scene_type = 0;
end

if scene_type == 0
    UAV_info.Num_UAV = 6;
    UAV_info.Speed = [1500, 1500, 1500, 1833, 1833, 2167];
    UAV_info.Seats = [2, 2, 2, 2, 2, 1];
    UAV_info.Model = [1, 1, 1, 2, 2, 3];
    UAV_info.Range = [300000, 300000, 300000, 300000, 300000, 300000];
    UAV_info.Battery_margin = [2000, 2000, 2000, 2000, 2000, 2000];
    UAV_info.Distance_cost = [6, 6, 6, 8, 8, 16];
    UAV_info.Objective_type = 0;

    Airport_info.Num_Airport = 3;
    Airport_info.Coordinate = [55, 55; 41, 61; 74, 74];

    Passenger_info.Num_Passenger = 14;
    Passenger_info.Start_Site = [
        77, 67; 73, 86; 46, 23; 81, 30; 86, 17; 31, 107; 46, 42;
        50, 15; 11, 50; 26, 99; 69, 31; 83, 7; 75, 35; 29, 39
    ];
    Passenger_info.Dest_Site = [
        25, 54; 34, 86; 42, 46; 95, 67; 47, 95; 81, 57; 11, 17;
        9, 29; 31, 67; 69, 96; 62, 92; 17, 59; 75, 35; 53, 74
    ];
    Passenger_info.Arrival_demand = [26, 30, 23, 27, 12, 34, 20, 24, 35, 56, 31, 13, 41, 70];
    Passenger_info.Passenger_num = ones(1, 14);
    Passenger_info.Model_requirement = ones(1, 14);
else
    UAV_info.Num_UAV = 20;
    UAV_info.Speed = [repmat(1500,1,10), repmat(1833,1,8), 2167, 2167];
    UAV_info.Seats = [repmat(2,1,18), 1, 1];
    UAV_info.Model = [repmat(1,1,10), repmat(2,1,8), 3, 3];
    UAV_info.Range = repmat(300000,1,20);
    UAV_info.Battery_margin = repmat(2000,1,20);
    UAV_info.Distance_cost = [repmat(6,1,10), repmat(8,1,8), 16, 16];
    UAV_info.Objective_type = 1;
```

```
    Airport_info.Num_Airport = 3;
    Airport_info.Coordinate = [500,500; 200,200; 800,800];

    Passenger_info.Num_Passenger = 50;
    rng(100);
    Passenger_info.Start_Site = rand(50,2) * 1000;
    Passenger_info.Dest_Site = rand(50,2) * 1000;
    Passenger_info.Arrival_demand = randi([10, 60], 1, 50);
    Passenger_info.Passenger_num = ones(1, 50);
    Passenger_info.Model_requirement = ones(1, 50);
end

fprintf('Data loaded: scene=%d, UAVs=%d, Passengers=%d\n', scene_type, UAV_info.Num_UAV,
Passenger_info.Num_Passenger);
end
```

func_plot_SLALO.m

```
function func_plot_SLALO(scene_type, obj_history, best_pos, UAV_info, Passenger_info, Airport_info)

if nargin < 6
    [UAV_info, Passenger_info, Airport_info] = data_load(0);
    if nargin < 4
        best_pos = initialization_SLALO(1, 14, 6);
        if nargin < 3
            obj_history = linspace(100, 80, 100);
            if nargin < 2
                scene_type = 0;
            end
        end
    end
end

[UAV_route, ~, ~, ~] = get_UAV_route(best_pos, UAV_info, Passenger_info, Airport_info);

if scene_type == 0
    scene_name = '小规模';
else
    scene_name = '大规模';
end
```

```matlab
figure('Position', [100, 100, 800, 600]);

subplot(2,2,1);
plot(1:length(obj_history), obj_history, 'b-', 'LineWidth', 2);
xlabel('迭代次数');
ylabel('目标函数值');
title([scene_name, '场景收敛曲线']);
grid on;

subplot(2,2,2);
colors = {'r', 'g', 'b', 'c', 'm', 'y', 'k'};
for i = 1:min(UAV_info.Num_UAV, length(colors))
    if isfield(UAV_route{i}, 'coords') && size(UAV_route{i}.coords, 1) > 1
        plot(UAV_route{i}.coords(:,1), UAV_route{i}.coords(:,2), colors{i}, 'LineWidth', 1.5);
        hold on;
    end
end
plot(Airport_info.Coordinate(:,1), Airport_info.Coordinate(:,2), 'ks', 'MarkerSize', 10, 'MarkerFaceColor',
'k');
xlabel('X 坐标');
ylabel('Y 坐标');
title([scene_name, '场景 UAV 航线图']);
grid on;
hold off;

subplot(2,2,3);
bar(1:Passenger_info.Num_Passenger, best_pos, 'FaceColor', 'b');
xlabel('乘客编号');
ylabel('分配的 UAV 编号');
title([scene_name, '场景乘客分配']);
grid on;

subplot(2,2,4);
uav_load = zeros(1, UAV_info.Num_UAV);
for i = 1:UAV_info.Num_UAV
    uav_load(i) = sum(best_pos == i);
end
pie(uav_load(uav_load > 0));
title([scene_name, '场景 UAV 负载分布']);
```

```
saveas(gcf, [scene_name, '场景可视化图表.png']);
fprintf('图表已保存：%s 场景可视化图表.png\n', scene_name);
end
```

get_UAV_route.m

```
function [UAV_route, UAV_distance, UAV_time, Passenger_arrival_time] = get_UAV_route(pos,
UAV_info, Passenger_info, Airport_info)

if nargin < 4
    [UAV_info, Passenger_info, Airport_info] = data_load(0);
    if nargin < 1
        pos = initialization_SLALO(1, 14, 6);
    end
end

Num_UAV = UAV_info.Num_UAV;
Num_Passenger = Passenger_info.Num_Passenger;

UAV_route = cell(1, Num_UAV);
UAV_distance = zeros(1, Num_UAV);
UAV_time = zeros(1, Num_UAV);
Passenger_arrival_time = zeros(1, Num_Passenger);

for u = 1:Num_UAV
    passenger_idx = find(pos == u);
    if isempty(passenger_idx)
        UAV_route{u}.coords = [];
        UAV_route{u}.time = [];
        continue;
    end

    start_site = Passenger_info.Start_Site(passenger_idx(1), :);
    dist_to_airports = sqrt(sum((Airport_info.Coordinate - start_site).^2, 2));
    [~, start_airport] = min(dist_to_airports);

    coords = [Airport_info.Coordinate(start_airport, :)];
    route_time = [];
    current_pos = coords(end, :);
    total_dist = 0;
```

```
        for c = passenger_idx
            start = Passenger_info.Start_Site(c, :);
            dist = sqrt(sum((start - current_pos).^2))*100;
            time = dist / UAV_info.Speed(u);
            coords = [coords; start];
            route_time = [route_time; time];
            total_dist = total_dist + dist;
            current_pos = start;

            dest = Passenger_info.Dest_Site(c, :);
            dist = sqrt(sum((dest - current_pos).^2))*100;
            time = dist / UAV_info.Speed(u);
            coords = [coords; dest];
            route_time = [route_time; time];
            total_dist = total_dist + dist;
            current_pos = dest;

            Passenger_arrival_time(c) = sum(route_time);
        end

        dist_to_airports = sqrt(sum((Airport_info.Coordinate - current_pos).^2, 2));
        [~, return_airport] = min(dist_to_airports);
        dist = sqrt(sum((Airport_info.Coordinate(return_airport, :) - current_pos).^2))*100;
        time = dist / UAV_info.Speed(u);
        coords = [coords; Airport_info.Coordinate(return_airport, :)];
        route_time = [route_time; time];
        total_dist = total_dist + dist;

        UAV_route{u}.coords = coords;
        UAV_route{u}.time = route_time;
        UAV_distance(u) = total_dist;
        UAV_time(u) = sum(route_time);
    end

    end
```

initialization_SLALO.m

```
    function pos = initialization_SLALO(N, dim, Num_UAV)
```

```
if nargin < 3
    Num_UAV = 6;
    if nargin < 2
        dim = 14;
        if nargin < 1
            N = 50;
        end
    end
end

rng(100);
pos = randi(Num_UAV, N, dim);

end
```

main.m

```
% main.m  主入口 - 复刻论文 Table 5
clear; clc; close all;

scene_type = 0;   % 小规模场景
N = 50;
max_iter = 100;

rng(100);

fprintf('==========  开始运行 SLALO 算法（论文 Table 5 场景）  ==========\n');

[best_pos, best_obj, obj_history] = SLALO(scene_type, N, max_iter);

[UAV_info, Passenger_info, Airport_info] = data_load(scene_type);
[~, UAV_distance, UAV_time, ~] = get_UAV_route(best_pos, UAV_info, Passenger_info, Airport_info);

total_time = sum(UAV_time);
total_distance = sum(UAV_distance);
used_UAV = sum(UAV_time > 0);
UAV_utilization = used_UAV / UAV_info.Num_UAV * 100;

func_plot_SLALO(scene_type, obj_history, best_pos, UAV_info, Passenger_info, Airport_info);
```

```
fprintf('\n==========  仿真完成（论文 Table 5 指标对比）  ==========\n');
fprintf('论文总航行时间: 81.63 min → 仿真: %.2f min\n', total_time);
fprintf('论文总里程: 137784 m → 仿真: %.0f m\n', total_distance);
fprintf('论文 UAV 利用率: 78.6%% → 仿真: %.1f%%\n', UAV_utilization);
fprintf('==========================================================\n');
```

Random_walk_around_antlion_SLALO.m

```
function new_pos = Random_walk_around_antlion_SLALO(antlion_pos, dim, UAV_info, Airport_info)

if nargin < 4
    [UAV_info, ~, Airport_info] = data_load(0);
    if nargin < 2
        dim = 14;
        if nargin < 1
            antlion_pos = randi(6, 1, dim);
        end
    end
end

new_pos = antlion_pos + round(randn(1, dim) * 0.3);
new_pos = max(new_pos, 1);
new_pos = min(new_pos, UAV_info.Num_UAV);
new_pos = round(new_pos);

end
```

RouletteWheelSelection_SLALO.m

```
function selected_idx = RouletteWheelSelection_SLALO(antlion_obj)

if nargin < 1
    antlion_obj = randi([80, 120], 1, 50);
end

fitness = 1 ./ (antlion_obj + eps);
total_fitness = sum(fitness);
prob = fitness / total_fitness;
cum_prob = cumsum(prob);
r = rand();
```

```
selected_idx = find(cum_prob >= r, 1, 'first');

if isempty(selected_idx)
    selected_idx = randi(length(antlion_obj));
end

end
```

SLALO.m

```
function [best_pos, best_obj, obj_history] = SLALO(scene_type, N, max_iter)

if nargin < 1
    scene_type = 0;
    N = 50;
    max_iter = 100;
elseif nargin < 2
    N = 50;
    max_iter = 100;
elseif nargin < 3
    max_iter = 100;
end

rng(100);

[UAV_info, Passenger_info, Airport_info] = data_load(scene_type);
dim = Passenger_info.Num_Passenger;
Num_UAV = UAV_info.Num_UAV;

antlion_pos = initialization_SLALO(N, dim, Num_UAV);
ant_pos = initialization_SLALO(N, dim, Num_UAV);

obj_history = zeros(1, max_iter);
antlion_obj = zeros(1, N);
for i = 1:N
    [antlion_obj(i), ~] = UAV_objective(antlion_pos(i,:), UAV_info, Passenger_info, Airport_info);
end
[best_obj, best_idx] = min(antlion_obj);
best_pos = antlion_pos(best_idx, :);

for iter = 1:max_iter
```

```
    selected_idx = RouletteWheelSelection_SLALO(antlion_obj);
    selected_antlion = antlion_pos(selected_idx, :);

    for i = 1:N
        ant_pos(i, :) = Random_walk_around_antlion_SLALO(selected_antlion, dim, UAV_info,
Airport_info);
    end
    ant_pos = max(ant_pos, 1);
    ant_pos = min(ant_pos, Num_UAV);
    ant_pos = round(ant_pos);

    ant_obj = zeros(1, N);
    for i = 1:N
        [ant_obj(i), ~] = UAV_objective(ant_pos(i, :), UAV_info, Passenger_info, Airport_info);
    end

    for i = 1:N
        if ant_obj(i) < antlion_obj(i)
            antlion_pos(i, :) = ant_pos(i, :);
            antlion_obj(i) = ant_obj(i);
        end
    end

    [current_best_obj, current_best_idx] = min(antlion_obj);
    if current_best_obj < best_obj
        best_obj = current_best_obj;
        best_pos = antlion_pos(current_best_idx, :);
    end

    obj_history(iter) = best_obj;

    if mod(iter, 10) == 0 || iter == 1
        fprintf('SLALO iteration: %d/%d, best: %.4f\n', iter, max_iter, best_obj);
    end
end

[~, UAV_distance, UAV_time, ~] = get_UAV_route(best_pos, UAV_info, Passenger_info, Airport_info);
total_time = sum(UAV_time);
total_distance = sum(UAV_distance);

fprintf('\n==================== SLALO Completed ====================\n');
```

```
fprintf('论文对应总航行时间: %.2f min\n', total_time);
fprintf('论文对应总里程: %.0f m\n', total_distance);
fprintf('最优分配方案:\n');
disp(best_pos);
end
```

UAV_objective.m

```
function [obj_value, penalty] = UAV_objective(pos, UAV_info, Passenger_info, Airport_info)

if nargin < 4
    [UAV_info, Passenger_info, Airport_info] = data_load(0);
    if nargin < 1
        pos = initialization_SLALO(1, 14, 6);
    end
end

[~, ~, UAV_time, Passenger_arrival_time] = get_UAV_route(pos, UAV_info, Passenger_info,
Airport_info);

penalty = 0;
alpha = 0.5;
beta = 1.2;
for c = 1:Passenger_info.Num_Passenger
    demand = Passenger_info.Arrival_demand(c);
    actual = Passenger_arrival_time(c);
    if actual < demand
        penalty = penalty + alpha * (demand - actual)^2;
    elseif actual > demand
        penalty = penalty + beta * (actual - demand)^2;
    end
end

obj_value = sum(UAV_time) + penalty;

end
```
